# Medical waste management in three areas of rural China

**DOI:** 10.1371/journal.pone.0200889

**Published:** 2018-07-20

**Authors:** Qiufeng Gao, Yaojiang Shi, Di Mo, Jingchun Nie, Meredith Yang, Scott Rozelle, Sean Sylvia

**Affiliations:** 1 Center for Experimental Economics in Education, Shaanxi Normal University, Xi’an, China; 2 Freeman Spogli Institute of International Studies, Stanford University, Stanford, California, United States of America; 3 Gillings School of Global Public Health, University of North Carolina at Chapel Hill, Chapel Hill, NC, United States of America; La Trobe University, AUSTRALIA

## Abstract

**Objective:**

The purpose of this paper is to describe current practices of medical waste management, including its generation, investments, collection, storage, segregation, and disposal, and to explore the level of support from upper tiers of the government and health care system for medical waste management in rural China.

**Methods:**

The authors draw on a dataset comprised of 209 randomly selected rural township health centers (THCs) in 21 counties in three provinces of China: Anhui, Shaanxi and Sichuan. Surveys were administered to health center administrators in sample THCs in June 2015.

**Results:**

The results show that the generation rate of medical waste was about 0.18 kg/bed, 0.15 kg/patient, or 0.13 kg/person per day on average. Such per capita levels are significant given China’s large rural population. Although investments of medical waste facilities and personnel in THCs have improved, results show that compliance with national regulations is low. For example, less than half of hazardous medical waste was packed in sealed containers or containers labeled with bio-hazard markings. None of the THCs segregated correctly according to the categories required by formal Chinese regulations. Many THCs reported improper disposal methods of medical waste. Our results also indicate low levels of staff training and low rates of centralized disposal in rural THCs.

**Conclusions:**

Medical waste is a serious environmental issue that is rising on the agenda of policymakers. While a large share of THCs has invested in medical waste facilities and personnel, it appears that actual compliance remains low. Using evidence of low rates of training and centralized disposal, we surmise that a lack of support from upper tiers of management is one contributing factor. Given these findings, we recommend that China’s policymakers should enhance support from upper tiers and improve monitoring as well as incentives in order to improve medical waste management.

## Introduction

Medical waste is a special category of waste because it poses both health and environmental risks [1–4]. In recent years, there has been increasing public awareness about the need to improve medical waste management [5,6]. Unfortunately, in many parts of the world, efforts to persuade governments that large investments are needed to improve medical waste management have been unsuccessful [7,8].

As the largest and one of the fastest-growing developing countries, China is facing important challenges with respect to the management of medical waste. Each year, the nation’s health system generates around 650,000 tons of medical waste. The quantity of medical waste is also growing rapidly, at an annual rate of around 20%, and the rise is occurring in both urban and rural areas [9,10]. Given that about half of China’s population lives in rural areas and that visits to rural primary health care institutions make up at least 38% of all health care visits in China, a large amount of medical waste is being generated in rural areas [11].

In recognition of the importance of medical waste management in both urban and rural areas, China’s government has enacted a number of national regulations that seek to address the control of medical waste. One of the main initiatives is the Medical Waste Control Act 380 (henceforth, Act 380) [12]. According to Act 380, the term “medical waste” is defined as any potentially hazardous solid waste that is generated by medical treatment facilities and/or laboratory facilities operating in a health center setting. Act 380, among other things, spells out how health facilities are supposed to treat medical waste and details the principles for collection, storage, segregation and disposal of waste. Following Act 380, the Ministry of Health and the State Environmental Protection Administration also issued Regulation 287 to establish categories for different types of medical waste [13]. Regulation 287 categorizes hazardous medical waste into five groups (infectious waste, pathologic waste, sharps waste, medicine waste and chemical waste).

Although Act 380 establishes regulations that are nationwide, there is currently limited evidence on compliance, particularly in rural areas where resources enabling compliance with national policy are limited. Researchers have begun to pay attention to medical waste management practices in China in recent years, mostly focusing on urban areas [3,9,14–20]. Recent papers find low compliance with medical waste management regulations in cities, but little is known about the state of medical waste management in rural China, especially for rural primary health care institutions (i.e. township health centers and village clinics). Of the few papers that do exist, to our knowledge, even fewer adopt quantitative methods to investigate medical waste in rural primary institutions [3,17]. In addition, the geographic coverage of each of these papers is limited to just one province. Considering the small number of studies and the unequal distribution of resources across different areas in China [21], current information is inadequate to comprehensively understand the implementation of medical waste management in rural China.

The overall goal of our study is to describe current practices of medical waste management and to explore the level of support from upper tiers of the government and health care system in rural areas of three provinces. To meet this goal, we have four specific objectives. First, using a dataset covering 21 counties in three provinces (one each from three of China’s main geographic regions), we aim to report the amount of medical waste generated from rural township health centers (henceforth, *THCs*). Second, we seek to examine medical waste investments in terms of the facilities and personnel of rural THCs. Third, we examine performance of medical waste collection, storage, segregation and disposal among rural THCs. Fourth, we explore the level of support from upper tiers of the government and health care system for management of medical waste in rural THCs.

The rest of the paper is organized as follows. Section two describes the sampling, data collection and variable definition. Section three reports results. Section four discusses the results. We conclude in section five.

## Sampling, data collection and variable definition

### Ethical approval

Approval from the institutional review boards of Stanford University, USA (protocol number: 25904) and Sichuan University, China (protocol number: K2015025) was obtained for this study. Consent from local authorities and sample THCs was obtained through direct communications with them. Before interviews with each of the directors from sample THCs, informed consent was obtained verbally from all providers participating in the study.

### Sampling

Our study focuses on rural THCs for two main reasons. First, as primary health care organizations in rural China, THCs are typically responsible for a large share of treatment for general diseases in rural communities [22]. Located in townships (which are mostly rural areas) and staffed by formally trained doctors and nurses, it is estimated that THCs serve more than 603 million people in rural China [11]. Second, since THCs make up the middle tier of China’s three-tiered rural health system (in between village clinics and county hospitals), outcomes at the THC level can also partially reflect problems in management and training by upper-level county hospitals [23].

The sample for this study was drawn from rural areas in three provinces: Sichuan, Shaanxi, and Anhui, which are located in Southwestern, Northwestern and Eastern China respectively. The THCs included in the study were selected from one prefecture (the administrative level below the province and above the county) in each of the three provinces, out of a total of 47 prefectures (10 in Shaanxi, 16 in Anhui, and 21 in Sichuan). The prefectures included in the study from each province were chosen for having a predominantly rural population and in consultation with local authorities.

The sample was selected to be representative of rural health systems in each of the three chosen prefectures using the following procedure. First, across the three prefectures, we randomly sampled 21 of 24 rural counties. Next, because even counties designated as “rural” have an urban township housing the county seat, we excluded the health center of the urban township. After excluding the urban townships, 10 townships were randomly sampled within each county and the primary THC of that township was included in the sample. A simple random sampling method was adopted to select the sample counties and townships. We first obtained lists of all the counties in the three prefectures. These counties constituted our sampling frame and we assigned a sequential number to each county. All possible counties were equally likely to be chosen. Then a random number generator was used to select the sample counties, using the sampling frame and sample size. With the same sampling method, we obtained our sample townships. Townships typically had only one THC. One county only had 9 rural townships, yielding a sample of 209 of the total 311 THCs in the 21 sample counties.

The average disposable income of rural residents in 2016 in the sample counties was close to the average of rural areas in each selected prefecture (in Sichuan, the average of sample counties was 10,773 RMB and the prefectural average was 12,834 RMB; in Anhui, the average of sample counties was 11,329 RMB and the prefectural average was 10,956 RMB; in Shaanxi, the average of sample counties was 10,340 RMB and the prefectural average was 10,582 RMB) [24]. The health facilities in the study’s sample counties serve a population of 9.9 million people, accounting for 73.8% of residents in the three prefectures [11].”

### Data collection

Data collection was carried out using a research team-designed questionnaire in June 2015. We collected information through in-depth interviews with staff members at each THC that were responsible for the management of medical waste. In most cases, we interviewed the Center Director of the THC. With few exceptions, all of the respondents had worked in the THC for more than five years and were accountable for the administration of their THC.

Detailed information was collected using a two-block survey. The questionnaire was designed based on national regulations for medical waste management (Act 380) and the WHO’s rapid assessment tool [4]. In the first block of the survey, specific questions were asked about the nature of the THC. When executing this part of the survey, enumerators asked the respondent to report data on THC human resources (e.g. the number of staff and number of doctors that work at the THC), THC physical resources (fixed assets, annual income, number of hospital beds) and patient demand for THC services (number of patients seen during a typical month, number of inpatients per month, bed occupancy rate).

In the second block of the survey, questions were asked specifically about medical waste investments, medical waste management practices and support from upper tiers of the health system. This block was designed mainly to collect data about the amount of medical waste generated; investments in medical waste management in terms of facilities and personnel; handling practices in collection, storage, segregation and final disposal of medical waste; and support from upper tiers in terms of medical waste training and centralized disposal management. Given the (potentially) somewhat sensitive nature of our questions, enumerators not only collected the data from the respondents using the survey instrument, they also carried out a physical inspection of the THC.

### Variable definition

To assess the amount of medical waste generated in each THC, enumerators asked interviewees to estimate (on average) the total amount of medical waste generated per week in kilograms. The generation rate (kg/bed per day; kg/patient per day; kg/person per day) for each THC was calculated using data on the number of available hospital beds, number of patients (including inpatients and outpatients), and number of persons (including patients and staff) reported in the first block of the survey.

To assess the general status of investments in medical waste management, enumerators asked interviewees about medical waste facilities and personnel. Specifically, questions were asked regarding whether there was a designated specialist to manage medical waste and whether there was a designated area to store medical waste in each THC. Enumerators asked to speak to the designated specialist responsible for medical waste and carried out an inspection to ensure that a real and qualifying storage area existed. Investments in personnel were also assessed by asking the designated specialist a series of questions on their awareness of the potential harms of improperly disposed medical waste.

To assess the general status of medical waste management outcomes, questions were asked about actual handling practices in terms of collection, storage, segregation and final disposal. Enumerators assessed collection and storage practices by first asking interviewees to guide them to the area where medical waste was collected and stored. Enumerators noted through observation whether medical waste had been stored according to Act 380 stipulations (e.g., whether medical waste was packed in sealed containers or containers with bio-hazard markings).

Segregation practices of medical waste were assessed by asking personnel to list the types of segregation categories used. This approach was used to judge whether THCs used the five categories for hazardous medical waste as stipulated in Regulation 287 (infectious waste, pathologic waste, sharp waste, medicine waste and chemical waste—see S1 Table). Enumerators further verified interviewee responses by physically checking for these reported categories in the storage area. In addition, interviewees were asked to describe the actual content materials in each of the categories they reported using. In our analysis, if we found that respondents segregated their trash differently than the rules stipulated, we marked their practices as incorrect. For example, if we found that needles were discarded with cotton balls and clothing in the category of infectious waste, this was interpreted as an incorrect segregation practice, as needles should be categorized as sharp waste. Based on these data, we calculated the number of segregation categories used in each THC and the number of THCs that practiced segregation correctly.

In terms of medical waste disposal, enumerators asked interviewees which methods were used to dispose of hazardous medical waste. Enumerators also physically inspected disposal areas in each THC. Based on the data, we calculated the number of THCs that reported using each disposal method.

To explore support from upper tiers, enumerators asked interviewees about their training in medical waste management and their use of centralized disposal. Questions were specifically asked about whether they had received medical waste training, whether they had received medical waste training in the past two years, and whether the THC used a centralized disposal method to handle hazardous medical waste. Act 380 states that all staff responsible for medical waste management and treatment should receive medical waste training. In addition, Act 380 also requires centralized disposal of hazardous medical waste. Centralized disposal typically refers to the practice of sending medical waste to a higher-level unit or centralized disposal company that is responsible for the final disposal. By law, THCs are not allowed to dispose of hazardous medical waste by any other methods.

## Results

According to the survey data, on average, each THC had about 25 staff members (Table 1, Row 1, Column 1). In general, there were about 7 doctors at each rural THC (Row 2, Column 1). The average value of fixed assets across THCs was 3.20 million RMB and the average annual income was 3.33 million RMB (Rows 3 & 4, Column 1). On average, each THC had 28.10 beds (Row 5). In general, about 1544 patients every month were seen at the typical THC; around four percent of the patients were inpatients (68 inpatients—Rows 6 & 7). The mean bed occupancy during our study period was about 49.70% (Row 8). The statistical differences in these characteristics among the three sample regions are reported in the S2 Table.

**Table 1:** Characteristics of sample township health centers.

Characteristics	Mean	SD	Min	P25 ^a^	P50 ^a^	P75 ^a^	Max
1. Number of staff ^b^	24.80	18.44	4.00	13.00	19.00	30.00	133.00
2. Number of doctors	7.64	7.15	1.00	3.00	5.00	9.00	53.00
3. Fixed assets (million) ^c^	3.20	4.18	0.25	1.10	2.20	3.79	42.00
4. Annual income (million)	3.33	3.22	0.40	1.38	2.27	4.02	30.50
5. Number of beds ^d^	28.10	32.83	4.00	12.00	20.00	30.00	400.00
6. Number of patients per month	1544.40	1406.61	25.33	653.33	1000.00	1866.67	7584.83
7. Number of inpatients per ^e^ month	67.55	75.94	0.00	19.25	43.83	85.67	500.00
8. Bed occupancy rate (%)	49.70	29.25	0.00	25.00	48.74	71.88	100.00

^a^ P25, P50, P75 refer to 25^th^ percentile, 50^th^ percentile and 75^th^ percentile respectively.

^b^ Staff in THCs includes doctors, nurses and other staff members such as management and custodial staff.

^c^ Fixed assets refers to the value of a THC’s medical equipment and its buildings.

^d^ Information from two THCs were omitted from our calculations of number of beds, inpatients per month, and bed occupancy rates as these hospitals did not provide overnight inpatient health services (meaning that there were no beds in their health centers).

^e^ In 2014, five of the THCs did not have inpatients and the bed occupancy rate was zero.

### Generation of medical waste

On average, each THC generated 3.90 kg of medical waste per day (Table 2, Row 1, Column 1). Adjusted for health center size, this means that medical waste is generated at a rate of 0.18 kg/bed per day (Row 2, Column 1). Each THC generated approximately 0.15 kg/patient and 0.13 kg/person per day (Rows 3 & 4, Column 1). The statistical differences in the medical waste generation rate among the three sample provinces can be seen in the S3 Table.

**Table 2:** Medical waste generation in sample township health centers.

Waste generation	Mean	SD	P25 ^a^	P50 ^a^	P75 ^a^
1. Total (kg/day)	3.90	6.87	0.83	1.82	3.57
2. Per bed (kg/bed per day)	0.18	0.34	0.05	0.09	0.17
3. Per patient (kg/patient per day) ^b^	0.15	0.45	0.03	0.05	0.11
4. Per person (kg/person per day) ^c^	0.13	0.34	0.02	0.05	0.11

^a^ P25, P50, P75 refer to 25^th^ percentile, 50^th^ percentile and 75^th^ percentile respectively.

^b^ The number of patients that we used includes number of inpatients and number of outpatients.

^c^ The number of persons that we used includes number of patients and number of staff.

### Medical waste investments in facilities and personnel

About three quarters of THCs (72%) in our sample had a designated area to store medical waste (Table 3, Row 1). The survey also shows that 77% of surveyed THCs have designated specialist personnel to collect and handle medical waste (Row 2).

**Table 3:** Medical waste facilities, specialist personnel and staff awareness in sample township health centers.

Variables	n = 209 (%)
1. Designated area to store waste (1 = yes) ^a^	151 (72)
2. Designated specialist to manage waste (1 = yes)	160 (77)
3. Staff awareness	
4. How harmful do you think medical waste is to the environment?	
Not harmful	2 (1)
A little harmful	12 (6)
Medium	20 (9)
Harmful	96 (46)
Very harmful	79 (38)
5. How serious is the improper disposal of medical waste?	
Not serious	2 (1)
A little serious	4 (2)
Serious	24 (11)
Very serious	179 (86)
6. Do you think you will be harmed if medical waste is not disposed of properly? (1 = yes)	164 (78)
7. Do you think local residents will be harmed if medical waste is not disposed of properly? (1 = yes)	175 (84)

^a^ A designated area to store waste must be sun-proof and rain-proof.

We assessed the level of awareness among medical staff on the importance of medical waste using four questions in our questionnaire (Table 3, Rows 3 to 7). More than 80% of our medical staff believed that medical waste would be harmful to the environment if improperly disposed of (Row 4). Almost all of the surveyed medical staff reported knowing that improper disposal of medical waste could lead to serious harm (Row 5). In fact, most of them indicated that improper disposal of medical waste would also endanger the health of local residents (Rows 6 & 7).

### Medical waste management practices in THCs

#### Collection and storage

We found that the majority (71%) of THCs reported packing their hazardous medical waste in containers (Table 4, Row 1). However, only 37% of all hazardous medical waste was packed in sealed containers, and only 49% of all hazardous medical waste was packed in containers labeled with bio-hazard markings (Rows 2 & 3).

**Table 4:** Medical waste collection, storage, segregation and disposal in sample township health centers.

Variables	n = 209 (%)
**Collection and storage**	
1. Packed in containers (1 = yes)	149 (71)
2. Packed in sealed containers (1 = yes)	78 (37)
3. Packed in containers with bio-hazard markings (1 = yes)	102 (49)
**Segregation categories** ^**a**^	
4. Non-hazardous waste	44 (21)
5. Infectious waste	109 (52)
6. Sharps waste	107 (51)
7. Medicine waste	40 (19)
8. Chemical waste	20 (10)
9. Pathologic waste	19 (9)
**Number of categories segregated into**	
10. Segregation into one type	48 (23)
11. Segregation into two types	55 (26)
12. Segregation into three types	60 (29)
13. Segregation into four types	32 (15)
14. Segregation into five types	12 (6)
15. Segregation into six types	2 (1)
16. Segregation done correctly ^**b**^	0 (0)
**Disposal** ^**c**^	
17. Burned	67 (32)
18. Burned and then put in landfill	36 (17)
19. Put in landfill	24 (11)
20. Disposal as household garbage	22 (11)
21. Sold to recycling vendors	2 (1)

^a^ Number and proportion of facilities reporting categories used in segregation of medical waste. We focus on six categories. In addition to hazardous medical waste (which should be segregated into five different categories, see S1 Table), the category of non-hazardous waste (typically disposed of with household garbage) is also included in this table.

^b^ The results were calculated by asking survey respondents to describe the type of materials that were discarded into each of the categories that they reported using. Responses were marked as correct if materials listed corresponded with correct segregation practices (by law); responses were marked as incorrect if not.

^c^ In addition, 51% of THCs reported using centralized disposal. According to our data, 36% of sample THCs used more than one type of disposal method.

#### Segregation of medical waste

In terms of segregation practices, only one fifth (21%) of THCs in our sample used the non-hazardous category to segregate medical waste (Table 4, Row 4). More than half of the surveyed THCs segregated their medical waste using the categories of infectious waste (52%) and sharp waste (51%—Rows 5 & 6). Less than one in every five THCs also used the category of medicine waste (19%—Row 7). Notably, less than 10% of our sample THCs segregated their medical waste into chemical waste (10%) or pathologic waste (9%) types (Rows 8 & 9).

In order to further understand segregation practices among sample THCs, we calculated the number of categories (or types of waste) that THCs used when segregating their medical waste (Table 4). Half of the surveyed THCs segregated their medical waste into two or less types of waste (49%—Rows 10 & 11). The rest of the THCs reported that their medical waste was divided into three (29%—Row 12) or four categories (15%—Row 13). Only seven percent of THCs segregated medical waste into five or more categories (Rows 14 & 15). None of the THCs in our sample segregated their medical waste correctly according to Chinese regulations (Table 4, Row 16).

#### Disposal of hazardous medical waste

In half of the sample THCs, medical personnel used burning as a method of disposing hazardous medical waste (Table 4, Rows 17 & 18). Slightly more than 10% of THCs reported dumping medical waste into landfills (Row 19). In some rural THCs, hazardous medical waste was disposed of jointly with household garbage (11%) or sold to recycling vendors (1%—Rows 20 & 21).

#### Support from upper tiers

According to our data, we find that about one-third (29%) of medical staff (specifically responsible for managing medical waste) had never received any kind of medical waste training and only around half (56%) of medical staff had received medical waste training in the past two years (Table 5, Row 1). Moreover, despite being mandated by the upper level government, only about half (51%) of the THCs disposed of hazardous medical waste through centralized disposal (Row 2).

**Table 5:** Support from upper tiers: medical waste training and centralized disposal of medical waste in sample township health centers.

Variables	n = 209 (%)
1. Have received medical waste training	
Never	61 (29)
Over two years ago	31 (15)
In the past two years	117 (56)
2. Used centralized disposal methods to handle medical waste (1 = yes)	106 (51)

## Discussion

In this paper, our main findings are fourfold. First, on average, each rural THC generated 3.90 kg of medical waste per day and the generation rate was about 0.18 kg/bed, 0.15 kg/patient or 0.13 kg/person per day in the sampled areas. Second, in terms of medical waste investments in facilities and personnel, about three quarters of sample THCs had designated storage areas and a similar percentage had specialist personnel to manage medical waste. Third, the main findings on medical waste management practices in THCs were that: a) less than half of the hazardous medical waste was packed in sealed containers or containers that were labeled with bio-hazard markings; b) none of the sample THCs segregated correctly according to Chinese regulations; and c) a large number of THCs still used improper disposal methods. Fourth, for training support and centralized disposal management from upper tiers, we find that 29% of medical staff had not received any medical waste training and 49% of THCs did not use centralized disposal methods.

The generation rate of medical waste found in THCs in rural China is comparably higher than those reported by other developing countries [4]. According to a WHO report, the generation rate of medical waste in primary health-care centers was 0.01–0.04 kg/patient per day in developing countries such as Pakistan and Tanzania. If we were to extrapolate our findings to other areas of rural China, the medical waste generated by rural village clinics and township health centers in China is more than 0.15 kg/patient per day. In addition, considering the large number of rural healthcare facilities (36,817 THCs and 640,536 village clinics in 2015) and the rural population (603 million in 2015) in China, the total amount of medical waste generated in rural areas annually is potentially huge [11]. In 2015, the total number of patient visits in rural THCs is 1.05 billion [25]. If we use the medical generation rate of 0.15 kg/patient per day to estimate the total amount of medical waste generated in rural THCs, it would be at least 0.16 million tons every year.

Despite investments and improvements in China’s medical waste management, we find that current medical waste collection, storage, segregation, and disposal practices among rural THCs are poor compared to national regulations. On the one hand, we find that around three quarters of rural THCs in our sample reported having designated storage areas or having designated personnel. In addition, the majority of staff members in the sample THCs acknowledged the serious risks of improper medical waste management, especially the risks on human health. However, less than half of hazardous medical waste was packed in sealed containers or packages that were labeled with bio-hazard markings. None of the sample THCs segregated correctly according to Chinese regulations, with many THCs reporting improper disposal methods. Using these metrics, this paper finds that medical waste management practices in rural China were much worse than those found in many other countries that with better developed waste treatment systems (e.g., Canada, France and Japan) [26–29]. In contrast, our findings are similar to those from other countries with younger waste treatment systems such as India and Brazil [30,31].

So why is it that, despite high investment into medical waste facilities and personnel, the outcomes of medical waste management in rural China are poor? While speculative, one potential reason is a lack of support from upper tiers of the health system. This is reflected in the low levels of training found among medical staff and low rates of centralized disposal. Viewed in this light, poor medical waste management practices may be a symptom of fragmentation in China’s health system [32]. Despite a series of health care reform policies in the last three decades, China’s health sector suffers from institutional fragmentation [32]. With more than ten government agencies involved in the health sector, each with its own set of objectives, the lack of coordination across institutional actors can be an obstacle to policy implementation [33]. In the case of medical waste management, responsibility is distributed across health bureaus, environmental bureaus, hospitals, and even centralized disposal centers. Such fragmentation can pose a challenge for the overall supervision and monitoring of medical waste management outcomes. Recent research has shown that multi-criteria decision making tools can help make effective decisions about medical waste treatment even in situations involving multiple stakeholders and complex evaluation criteria; such tools may be useful in helping to address the fragmentation problem [34–37].

Under the decentralized health system, primary health care institutions are left largely dependent on local governments who often have fewer financial resources and support [38–40]. While the central government may set the overall policy, in many cases, enforcement of policies depends mainly or entirely on local funding [41]. Frequently, particularly for more remote and rural hospitals, this funding is not sufficient. In one study conducted in the city of Xinxiang, Henan province, it was found that local medical institutions received little support in terms of transport costs for medical waste management from upper tiers of government to sustain their costs [16]. In fact, financial support was lacking to the point that many hospitals could not afford to transport their medical waste off-site, despite national requirements that they do so. At the same time, the relatively small quantities of medical waste produced by rural or remote hospitals provided little incentive for disposal centers to service these areas to pick up waste [14,16]. Without off-site treatment of medical waste, hospitals had even less of an incentive to enforce proper segregation, packing or disposal of waste.

This study makes important contributions to medical waste management research in three main ways. First, to our knowledge, our paper is the first large, cross-sectional study to investigate medical waste management in rural areas of China. Second, our quantitative data includes a rich set of information regarding the generation, investments, collection, storage, segregation, and disposal of medical waste, allowing us to compare the status of medical waste management in rural China with other places on an international scale. Third, our research also allows us to comment on the degree of upper-tier support provided in the form of centralized disposal channels or medical waste training, offering key insights into possible factors that contribute to low compliance with medical waste management regulations in rural China.

This study has several limitations. First, our study sample is only drawn from three regions and is not necessarily representative of rural THCs nationally. Yet, we sample from three different regions, while previous studies only focus on one region in China. Second, in this study, we mainly rely on responses from THC staff. We try to mitigate bias in survey responses by confirming with observation. Future studies could go further by taking samples of medical waste to confirm representativeness and by seeking to adopt multiple measures to reduce bias in the investigation.

## Conclusion

The generation rate of medical waste in the surveyed THCs in rural China was about 0.18 kg/bed, 0.15 kg/patient or 0.13 kg/person per day on average. Rural THCs were found to have low rates of compliance with national regulations in terms of medical waste collection, storage, segregation, and disposal management. Our findings suggest that there is a need for greater policy attention on the proper management of medical waste. Beyond investments into equipment, facilities and specialist personnel, effective approaches to improving waste management practices will likely entail a greater degree of support from upper tiers of government in the form of quality training and coordination of centralized disposal. Effective policy will also require an improved process for monitoring compliance with regulations, as well as incentives for facilities to comply with regulations.

## Supporting information

S1 Table Classification of hazardous medical waste (China Ministry of Health, 2003). (DOCX)

S2 Table Statistical comparisons of median values of the characteristics of sample township health centers. (DOCX)

S3 Table Statistical comparisons of median values of medical waste generation in sample township health centers. (DOCX)

S4 Table Original survey questionnaire used in the study. (DOCX)
