# Supplementary material for: Medical waste management in three areas of rural China
Source: PLoS One. 2018 Jul 20;13(7):e0200889. doi: 10.1371/journal.pone.0200889 (PMC6054418; doi:10.1371/journal.pone.0200889)
Supplement: S1 Table — (DOCX) [file pone.0200889.s001.docx]

**S1 Table. Classification of hazardous medical waste (China Ministry of Health, 2003)**

| **Category** | **Descriptions** | **Examples** |
| --- | --- | --- |
| Infectious waste | Waste suspected to contain a variety of pathogens that may lead to the spread of infectious diseases | Items contaminated by patients’ blood, body fluids and excretions (e.g. cotton balls, discarded clothing); waste and other materials that have been in contact with patients infected with highly infectious diseases in isolation wards or suspected patients admitted by medical institutions; laboratory cultures and microbiological stocks; used medical specimens; waste contaminated with blood and other body fluids; used single-use disposable medical supplies |
| Pathologic waste | Human waste and animal pathological waste from treatment, diagnosis and/or medical experiments | Human or animal pathological waste, including tissues, organs or fluids, body parts |
| Sharps waste | Used or unused medical sharps waste that could cause a cut or puncture | Needles, syringes, blades, scalpels, broken glass (e.g. used slides, glass vials, etc.) and other items that could cause a cut or puncture |
| Medicine waste | Expired, discarded, obsolete, contaminated or no longer required common medicines | Common medicines that are expired, discarded or are no longer required; cytotoxic waste containing substances with genotoxic properties (e.g. waste containing cytostatic drugs – often used in cancer therapy; genotoxic chemicals); discarded vaccine products and blood products |
| Chemical waste | Toxic, corrosive, flammable and explosive chemicals | Waste containing chemical substances (e.g. laboratory reagents); disinfectants that are expired or are no longer needed (e.g. peracetic acid); waste with high content of heavy metals (e.g. blood-pressure gauges and broken thermometers) |
