# Supplementary material for: Medical waste management in three areas of rural China
Source: PLoS One. 2018 Jul 20;13(7):e0200889. doi: 10.1371/journal.pone.0200889 (PMC6054418; doi:10.1371/journal.pone.0200889)
Supplement: S2 Table — (DOCX) [file pone.0200889.s002.docx]

**S2 Table. Statistical comparisons of median values of the characteristics of sample township health centers**

| **Characteristics** | **Shaanxi Province (n=80)** | | **Sichuan Province (n=70)** | | **Anhui Province (n=59)** | | **F (P) ^a^** |
| --- | --- | --- | --- | --- | --- | --- | --- |
|  | **Mean** | **SD** | **Mean** | **SD** | **Mean** | **SD** |  |
| 1. Number of staff ^b^ | 15.45 | 8.69 | 31.74 | 23.82 | 29.25 | 15.58 | 20.08 (0.000) |
| 2. Number of doctors | 4.74 | 5.71 | 9.19 | 8.22 | 9.75 | 6.30 | 11.91 (0.000) |
| 3 .Fixed assets (million) ^c^ | 1.65 | 1.77 | 4.00 | 4.33 | 4.35 | 5.54 | 9.79 (0.000) |
| 4. Annual income (mullion) | 1.77 | 1.11 | 4.44 | 4.52 | 4.15 | 2.37 | 18.01 (0.000) |
| 5. Number of beds ^d^ | 17.00 | 9.53 | 39.77 | 51.07 | 29.12 | 16.61 | 9.73 (0.000) |
| 6. Number of patients per month | 902.97 | 871.41 | 1796.39 | 1546.30 | 2115.15 | 1497.05 | 16.42 (0.000) |
| 7. Number of inpatients per month | 41.01 | 37.16 | 112.36 | 104.90 | 49.63 | 43.16 | 22.52 (0.000) |
| 8.Bed occupancy rate (%) | 47.44 | 29.39 | 58.76 | 28.39 | 41.85 | 27.65 | 5.96 (0.003) |

^a^ Testing null hypothesis of no differences among the three sample provinces by F test (one-way ANOVA test) for means comparison.

^b^ Staff in THCs includes doctors, nurses and other staff members for management and custodial staff.

^c^ Fixed assets refer to the value of a THC’s medical equipment and its buildings.

^d^ Information from two THCs are omitted from our calculations of number of inpatients per month, number of beds, and bed occupancy rates as these hospitals did not provide overnight inpatient health services (meaning that there were no beds in their health centers).
