# Supplementary material for: Medical waste management in three areas of rural China
Source: PLoS One. 2018 Jul 20;13(7):e0200889. doi: 10.1371/journal.pone.0200889 (PMC6054418; doi:10.1371/journal.pone.0200889)
Supplement: S3 Table — (DOCX) [file pone.0200889.s003.docx]

**S3 Table. Statistical comparisons of median values of medical waste generation in sample township health centers**

| **Waste generation** | **Shaanxi province (n=80)** | | **Sichuan province (n=70)** | | **Anhui province (n=59)** | | **F (P) ^a^** |
| --- | --- | --- | --- | --- | --- | --- | --- |
|  | **Mean** | **SD** | **Mean** | **SD** | **Mean** | **SD** |  |
| 1. Total (kg/day) | 2.06 | 2.33 | 3.92 | 5.49 | 6.38 | 10.73 | 7.09 (0.001) |
| 2. Per bed (kg/bed per day) | 0.13 | 0.12 | 0.14 | 0.15 | 0.30 | 0.59 | 5.11 (0.007) |
| 3. Per patient (kg/patient per day) ^b^ | 0.23 | 0.68 | 0.08 | 0.07 | 0.13 | 0.25 | 2.06 (0.131) |
| 4. Per person (kg/person per day) ^c^ | 0.19 | 0.51 | 0.08 | 0.07 | 0.12 | 0.22 | 1.97 (0.142) |

^a^ Testing null hypothesis of no differences among the three provinces by F test (one-way ANOVA test) for means comparison

^b^ The number of patients that we used includes number of inpatients and number of outpatients.

^c^ The number of persons that we used includes number of patients and number of staff.
