# Supplementary material for: Medical waste management in three areas of rural China
Source: PLoS One. 2018 Jul 20;13(7):e0200889. doi: 10.1371/journal.pone.0200889 (PMC6054418; doi:10.1371/journal.pone.0200889)
Supplement: S4 Table — (DOCX) [file pone.0200889.s004.docx]

**S4 Table. Original survey questionnaire used in the study**

| **Seq.** | **Question 问题** | **Units/Options 单位/选项** |
| --- | --- | --- |
|  |  |  |
| 1 | Code | Please describe in words |
|  | 问卷编码 | 请文字描述 |
|  |  |  |
| 2 | 访谈日期 | Please describe in words |
|  | Survey date | 请文字描述 |
|  |  |  |
| 3 | Sample province | Please describe in words |
|  | 样本省 | 请文字描述 |
|  |  |  |
| 4 | Sample prefecture | Please describe in words |
|  | 样本市 | 请文字描述 |
|  |  |  |
| 5 | Name of the THC | Please describe in words |
|  | 乡镇卫生院名称 | 请文字描述 |
|  |  |  |
| 6 | Total number of staff | number of people |
|  | 乡镇卫生院人员总数 | 人数 |
|  |  |  |
| 7 | Total number of doctors | number of people |
|  | 乡镇卫生院医生总人数 | 人数 |
|  |  |  |
| 8 | Fixed assets of the THC (including medical equipment and the buildings) | yuan |
|  | 乡镇卫生院固定资产价值 (包括医疗设备和房屋建筑物) | 元 |
|  |  |  |
| 9 | Gross annual income of 2014 | yuan |
|  | 2014年全年总收入 | 元 |
|  |  |  |
| 10 | How many available beds in the THC | number of beds |
|  | 实际开放住院床位数 | 床 |
|  |  |  |
| 11 | Number of total patients in 2014 | person-time |
|  | 2014年全年门诊人次数 | 人次 |
|  |  |  |
| 12 | Number of total inpatients in 2014 | person-time |
|  | 2014年全年住院病人次数 | 人次 |
|  |  |  |
| 13 | The percentage of utilization of beds in 2014 | % |
|  | 2014年病床使用率 | % |
|  |  |  |
| 14 | Is there a designated specialist to manage medical waste in the THC? | 1=Yes，2=No |
|  | 乡镇卫生院是否有专人负责医疗废物的管理 | 1=是，2=否 |
|  |  |  |
| 15 | Is there a designated area to store medical waste (must be sun-proof and rain-proof)? | 1=Yes，2=No |
|  | 是否与专用场所存放医疗废物（防晒、防雨淋） | 1=是，2=否 |
|  |  |  |
| 16 | Have you ever received medical waste training? | 1=Yes，2=No |
|  | 您是否参加过医疗废物相关的培训 | 1=是，2=否 |
|  |  |  |
| 17 | When was the last time you participated in medical waste training? | year |
|  | 您最后一次参加医疗废物培训的时间 | 年 |
|  |  |  |
| 18 | Please describe all types of medical waste generated in the THC | Please describe in words |
|  | 请描述乡镇卫生院产生的医疗废物有哪些 | 请文字描述 |
|  |  |  |
| 19 | On average, what is the total amount of medical waste generated per week in the THC in 2014? | kg |
|  | 2014年乡镇卫生院平均每周产生多少量的医疗废物 | 千克 |
|  |  |  |
| 20 | What types of medical waste categories does the THC use to segregate the generated medical waste? | a. Household waste 生活垃圾 |
|  |  | b. Infectious waste 感染性废物 |
|  |  | c. Pathologic waste 病理性废物 |
|  | 乡镇卫生院使用哪些分类条目分类产生的医疗废物 | d. Sharps waste 损伤性废物 |
|  |  | e. Medicine waste 药物性废物 |
|  |  | f. Chemical waste 化学性废物 |
|  |  | g. Other, please specify 其他，请说明 |
|  |  |  |
| 21 | Please list the types of medical waste that were included in each of the medical waste categories (used by the THC): |  |
|  | 乡镇卫生院使用的分类条目中，每类条目包含医院产生的哪些医疗废物: |  |
|  | a. Household waste | Please describe in words |
|  | 生活垃圾 | 请文字描述 |
|  | b. Infectious waste | Please describe in words |
|  | 感染性废物 | 请文字描述 |
|  | c. Pathologic waste | Please describe in words |
|  | 病理性废物 | 请文字描述 |
|  | d. Sharps waste | Please describe in words |
|  | 损伤性废物 | 请文字描述 |
|  | e. Medicine waste | Please describe in words |
|  | 药物性废物 | 请文字描述 |
|  | f. Chemical waste | Please describe in words |
|  | 化学性废物 | 请文字描述 |
|  | g. Other, please specify | Please describe in words |
|  | 其他，请说明 | 请文字描述 |
|  |  |  |
| 22 | Whether the medical waste was packed in special containers | 1=Yes (Please describe what kind of medical waste was packed)； 2=No |
|  | 医疗废物是否有专用的包装物或包装容器 | 1=有（请描述存放了哪些医疗废物）; 2=没有 |
|  |  |  |
| 23 | Whether the medical waste was packed in sealed containers | 1=Yes (Please describe which kind of medical waste was packed)； 2=No |
|  | 专用的包装物或包装容器是否密封 | 1=有（请描述存放了哪些医疗废物）; 2=没有 |
|  |  |  |
| 24 | Whether the medical waste was packed in containers with bio-hazard markings | 1=Yes，2=No |
|  | 专用的包装容器是否有警示标识和警示说明 | 1=是，2=否 |
|  |  |  |
| 25 | Which kind of disposal methods was used to handle the generated medical waste? | (Please describe the disposal methods used and the types of medical waste disposed of using that method) |
|  | 使用何种处理方式处理医疗废物 | 请描述每种处理方式处理的医疗废物种类 |
|  |  |  |
|  | *Staff awareness of medical waste management* |  |
|  | *管理人员意识* |  |
|  |  |  |
| 26 | How harmful do you think medical waste is to the environment? | 1=Not harmful; 2=A little harmful; 3=Medium; 4=Harmful; 5=Very harmful |
|  | 您认为医疗废物对环境的伤害程度是怎样的 | 1=没有伤害；2=伤害较小；3=伤害一般；4=伤害比较大；5=伤害非常大 |
|  |  |  |
| 27 | How serious is the improper disposal of medical waste? | 1=Not serious; 2=A little serious; 3=Serious; 4=Very serious |
|  | 您认为医疗废物未妥善处理的后果严重吗 | 1=后果不严重; 2=后果较小; 3=后果较大; 4=后果严重 |
|  |  |  |
| 28 | Do you think you will be harmed if medical waste is not disposed of properly? | 1=Yes，2=No |
|  | 您认为自己会因医疗废物未妥善处理而受到伤害吗 | 1=是，2=否 |
|  |  |  |
| 29 | Do you think local residents will be harmed if medical waste is not disposed of properly? | 1=Yes，2=No |
|  | 您认为村民会因医疗废物未妥善处理而受到伤害吗 | 1=是，2=否 |
